# Objective Measures of Emotion Related to Brand Attitude: A New Way to Quantify Emotion-Related Aspects Relevant to Marketing

**DOI:** 10.1371/journal.pone.0026782

**Published:** 2011-11-02

**Authors:** Peter Walla, Gerhard Brenner, Monika Koller

**Affiliations:** 1 School of Psychology, Faculty of Science and Information Technology, University of Newcastle, Newcastle, New South Wales, Australia; 2 Biological Psychology Unit, Faculty of Psychology, University of Vienna, Vienna, Austria; 3 Institute for Marketing Management, Vienna University of Economics and Business, Vienna, Austria; 4 Neuroconsult e.U., Vienna, Austria; University of Manchester, United Kingdom

## Abstract

With this study we wanted to test the hypothesis that individual like and dislike as occurring in relation to brand attitude can be objectively assessed. First, individuals rated common brands with respect to subjective preference. Then, they volunteered in an experiment during which their most liked and disliked brand names were visually presented while three different objective measures were taken. Participant's eye blinks as responses to acoustic startle probes were registered with electromyography (EMG) (i) and their skin conductance (ii) and their heart rate (iii) were recorded. We found significantly reduced eye blink amplitudes related to liked brand names compared to disliked brand names. This finding suggests that visual perception of liked brand names elicits higher degrees of pleasantness, more positive emotion and approach-oriented motivation than visual perception of disliked brand names. Also, skin conductance and heart rate were both reduced in case of liked versus disliked brand names. We conclude that all our physiological measures highlight emotion-related differences depending on the like and dislike toward individual brands. We suggest that objective measures should be used more frequently to quantify emotion-related aspects of brand attitude. In particular, there might be potential interest to introduce startle reflex modulation to measure emotion-related impact during product development, product design and various further fields relevant to marketing. Our findings are discussed in relation to the idea that self reported measures are most often cognitively polluted.

## Introduction

### 1. Background

These days, our environment not only provides us with stimuli that have to be evaluated to directly protect the conservation of oneself in order to survive. Instead, a lot of stimuli out there are not critically important, but still do play a certain role in our everyday life. Brand names are good examples for such stimuli. They stand for products sold under their names. They are associated with knowledge about respective products, with experience related to companies producing and/or selling them and supposedly they are also associated with emotions altogether forming the basis for individual brand attitude. From a company's perspective, creating a positive brand attitude is of utmost importance. The rationale for this circumstance is twofold. First, the attitude towards an object affects individual object-related behaviour [1]. Hence, positive attitudes towards a brand are likely to have a positive impact on purchase behaviour and brand loyalty. Second, the strategy to promote positive affective responses to a brand can increase its value which in turn is the basis for high brand equity and brand profitability [2], [3]. In the long run, consumer attitudes towards a brand can significantly shape a firm's economical performance. Given its predominant value for explaining consumer behaviour and brand-related issues, any research about brand attitude has always ranked high on the agenda. In the majority of studies, brand attitude either served as antecedent to purchase intention [4], [5] or as dependent variable when testing for various effects in advertising [2], [6].

However, given the vast amount of research including brand attitude to explain and predict other phenomena, there is a surprisingly small amount of conceptual and empirical discussions on the concept of brand attitude itself. Brand attitude comprises cognitive aspects in terms of brand associations [7] as well as a strong emotional component [8]. It encompasses the extent to which a firm is able to create emotional ties with the customer [9]. In 2006 [10], it was demonstrated that culturally familiar brands elicit prefrontal cortex activity. This finding could already be taken as objective physiological evidence that emotion plays a big role with respect to brand attitude, because among various other functions the prefrontal cortex is involved in emotion-related information processing. Brand attitude has traditionally been measured by rating or evaluative semantic differential scales [11]–[13]. These types of measures indicate the respondent's attitude towards the brand after a cognitive evaluation procedure. Although these measures are capable of capturing cognitive aspects like brand associations and informational aspects stored in memory, they fail to fully tap into the motivational and emotional facet of brand attitude.

### 2. Cognition and Emotion

Although largely viewed as separate functions, emotion and cognition are more and more considered interdependent. More precisely, it is believed that they influence each other while their merged output drives decision making and finally behaviour [14]. It was argued that at some point of information processing in the human brain, functional specialisation is lost whereat emotion and cognition conjointly contribute to the control of thought and behaviour [15]. This is where it usually gets difficult to track down only one or the other by using traditional methods. Nevertheless, it is assumed for the purpose of the present study that before that point arrives both functions provide their separate contributions to information processing and it must be possible to define them separately. Cognition-related aspects may be defined easier by traditional approaches because cognition is more tightly linked with language information processing. On the other hand, emotion is rather vague and abstract and therefore more difficult to describe. There might even be a hemispheric difference separating these two major functions. Cognition as a language-related type of evaluation may be processed predominantly by the left hemisphere while emotion is not primarily tied to language and rather processed by the right hemisphere. Only recently, neuroeconomics as an interdisciplinary blend of applying methods from neuroscience to resolve questions in marketing research, has been able to address the different roles of emotion and cognition in purchase decision-making in more detail [16]. The present study now evaluates and introduces an objective method known to be sensitive to emotion-related aspects of a stimulus, a method called startle reflex modulation.

### 3. Startle reflex modulation

Applied Neuroscience offers a number of ways to focus on emotion-related processing in the human brain. Besides common brain imaging methods such as electroencephalography (EEG) [17], [18], magnetoencephalography (MEG) [19]–[21], functional Magnet Resonance Imaging (fMRI) [22] and Positron Emission Tomography (PET) [23], startle reflex modulation is known to be a measure of emotion in terms of motivational aspects (valence; approach or avoidance) without demanding explicit responses [24], [25]. In the late 1980s, after many pioneer investigations in rodents it was found that humans demonstrate a modulated startle reflex as a function of emotional valence as reflected in degrees of pleasantness [26]. Since then, the magnitude of an eye blink as a response to loud and short acoustic white noise was mostly taken as a measure of startle reflex strength. Strikingly, eye blink amplitude is reduced in case of positive emotion (pleasant) whereas it is increased in case of negative emotion (unpleasant) [27]. It was even shown that rapid changes in emotion valence (or pleasantness) related to so-called lead stimuli (foreground emotional stimuli a participant is exposed to) are reflected in eye blink amplitudes as startle responses [28], [29]. For example, random presentations of different facial expressions led to corresponding modulations of eye blink amplitudes [30], [31].

Besides the objective nature of this method (no language-related explicit responses have to be given by study participants) the crucial advantage of startle reflex modulation is its independence from cognitive information processing and from arousal. It was demonstrated in 1988 [26] that only startle responses followed the pattern of study participant's self report about emotional valence, whereas arousal, interest, viewing time and skin conductance data did not allow to distinguish between negative and positive emotional valence. Despite some arousal-related influences that occur, the main sensitivity of startle responses is in the valence domain. They increase with negative valence and decrease with positive valence. Skin conductance though, indicating sweat gland activity, increases with higher levels of arousal independent of emotional valence. Thus, modulated startle responses rather reflect changes in the valence dimension whereas different skin conductance values reflect different levels of arousal.

The fact that startle responses quickly adapt to changing emotion context together with its independency from cognitive influence makes it an ideal tool to quantify emotion (with respect to motivational aspects) on a fine graded scale.

We therefore formed the hypothesis that ongoing visual perception of strongly liked brand names elicits less pronounced startle responses compared to ongoing visual perception of strongly disliked brand names. According to the above mentioned features of startle responses we further link any such significant findings with differences in emotion-related (valence) information processing initiated during visual perception of known brand names.

### 4. Additional Psychophysiology

Besides startle reflex modulation, two traditional measures in psychophysiology, skin conductance and heart rate, are also known to be sensitive to certain aspects of emotion [32]. However, these measures are more vulnerable to other factors such as arousal than is startle reflex modulation. Nevertheless, they provide further insight into physiological alterations in response to emotion-related stimulation and were therefore chosen in addition to startle reflex modulation in the present study.

The hypothesis here is that both heart rate and skin conductance differ significantly while being recorded during ongoing visual perception of written brand names as a function of prior stated subjective preference.

## Methods

### Ethics statement

This study was conducted by using non-invasive methods, thus no local ethics committee was needed to carry it out at our institution. However, it was ethically approved by the head and by the deputy head of the Biological Psychology Unit of the Faculty of Psychology. In addition, written informed consent was given by all participants who could withdraw at any time during the experiment without any negative consequences.

### 1. Participants

29 young and healthy study participants volunteered in the present study. 8 participants had to be excluded from the analysis because of unwanted muscle-artefacts, no clear startle responses or too many missing values. The mean age of the remaining 21 participants (14 Females) was 28.14 (SD = 6.29). They were all right handed and with normal or corrected to normal vision. All volunteers were paid for participation which was possible due to a generous financial support by the Austrian Bank *Bank Austria* (see acknowledgements). They all gave their written informed consent.

### 2. Stimuli

300 common brand names (common in German speaking countries) were put together on a list on the basis of subjective selection by the experimenters. These brand names were all presented through an online survey using the EFS Survey solution offered by Globalpark (www.globalpark.com). This online survey allowed study participants to rate all brand names according to individual attitude (like or dislike). For this purpose each brand name was simultaneously presented with a slider scale ranging from 1 to 21 (almost analogue). A bar could be placed at one of the fine grades between the extremes by using a computer mouse. The left side was meant to rate for dislike-related intensity while the right side was meant to rate for like-related intensity. As a consequence of this pre-evaluation we were able to identify the 10 most liked and the 10 most disliked brand names out of the entire list for every participant (individual extreme attitude ratings). These individual sets of 20 brand names were then introduced as independent variables (target brands) in a following startle reflex modulation experiment which also included measures of skin conductance and heart rate. For every participant a separate list was created including individual target brand names mixed up with 180 non-target brand names (filler items). The order of target and non-target brand names was randomised between participants.

### 3. Physiological measurements

#### 3.1. Startle Reflex

Eye blinks as startle responses were elicited by a 50 ms burst of acoustic white noise at 105 dB sound pressure level delivered through professional headphones (AKG) fully covering both ears. Sound pressure level was measured with a mobile measuring device (produced by Voltcraft). To achieve the respective loudness a commercial headphone pre-amplifier was used (Behringer; MicroAMP HA400). With bipolar electromyography (EMG) carried out with a Nexus-10 mobile recording system (by Mind Media BV) muscle potential changes of the *musculus orbicularis oculi* of the left eye of every study participant were measured and stored on the hard drive of a lab top computer. We used a dual channel electrode cable with carbon coating and active shielding technology for low noise and an additional ground electrode cable. EMG sampling rate was 2048 per s. A band pass filter from 20 Hz to 500 Hz was applied during online recording. Raw EMG data were then recalculated by using the root mean square (RMS) method to transform EMG signals into amplitudes. The resulting amplitudes were then subject to statistical analysis.

#### 3.2. Skin conductance and heart rate

Skin conductance was recorded at a rate of 32 samples/s with a Nexus-10-SC/GSR sensor (Two finger sensor) connected to the Nexus-10 recording system with a 24 bit resolution which is able to register changes of less than 0.0001 microsiemens. We attached one sensor to the middle finger and the other sensor to the ring finger of the left hand.

Heart rate was calculated from the raw blood volume pulse signal recorded at a rate of 32samples/s with a Nexus-10-Blood Volume Pulse sensor. This sensor works with near infrared light, a method known as photoplethysmography. It was also connected to the Nexus-10 recording system and attached to the left index finger.

### 4. Procedure

After arriving at the lab every participant was seated on a comfortable chair viewing a computer monitor which was placed in front of them on a desk. It was all set up in a specially designed experiment room with a computer monitor on a table and one chair. A door could be closed to leave each study participant alone to fully concentrate on the task. All stimulus presentation and physiological signal recording could be controlled from outside. The procedure was explained to the participants while all sensors were attached, the cables connected to the recording system and all computers and monitors turned on. To visually present the individually prepared stimulus lists we used the software Biotrace+ (Mind Media BV). Each brand name (white letters on black background) was presented for 5 s with an inter stimulus interval of 2 s. Between stimuli a black screen with a fixation cross in the centre was shown. As mentioned above, stimulus lists were created individually based on each subject's online rating. Thus, lists differed between subjects. The 180 filler brand names were randomly mixed with the 20 individual brand names for every subject. All 20 target brand names were associated with a startle probe at 4.2 s after their onset. Inter stimulus interval between startle probes was kept between 40 s and 70 s to avoid unwanted startle habituation effects. This was done although it was demonstrated that the modulatory influence of the startle reflex is much less subject to habituation than is the obligatory startle pathway [28]. In other words, startle responses keep their sensitivity to reflect emotion valence even if they habituate with respect to startle stimulation as such.

The instruction given to study participants was to read each brand name and to rate their buying intention on a five-graded scale. This instruction was simply used to keep our participant's conscious attention focused on the brand names. All participants were naive to the nature of the experiment before being contacted, but they were fully informed shortly before actual testing.

BioTrace+ was also used to manage data registration and following data pre processing before statistical analysis.

### 5. Data processing

#### 5.1. Startle reflex data

**Figure 1:**
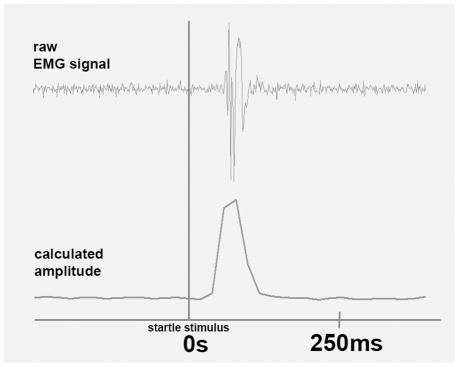
Electromyography: This figure shows the transfer from a raw EMG signal into an amplitude signal. Amplitude peaks which reflect eye blink magnitude were subject to statistical analysis.

All raw EMG signals (changes in potential) following startle stimulations (20 per participant) were recalculated into amplitude signals by using the root mean square method (RMS) for all study participants (as implemented in the software Biotrace+) (figure 1). For computing RMS an epoch size of 1/16 s was used. The transformation of raw EMG signals into amplitude signals is common practise within startle response modulation research, because an amplitude signal is simply one number to calculate with reflecting strength of muscle construction. All resulting relative eye blink amplitudes were visually inspected. In case of obviously bad signal-to-noise ratios resulting in no visible signal amplitudes respective data were not taken into account for further analysis. Individual data were only entered into statistical analysis if at least 6 out of a maximum of 10 amplitudes for each of the two conditions were evident (liked and disliked brand names). Only a few missing values occurred in 5 subjects. These missing values were replaced by means of existing values from each respective condition. The final data set included 10 single eye blink amplitudes for liked and further 10 single eye blink amplitudes for disliked brand names per participant. Finally, these eye blink amplitudes were subject to statistical analysis in terms of repeated measures ANOVA to calculate condition main effect significance. Effect sizes were also calculated to better interpret statistical findings.

#### 5.2. Skin conductance and heart rate data

Mean skin conductance values (in microsiemens) as well as mean heart rate values (in beats per minute) were calculated for time windows starting from the onset of brand name presentation until 4 s after the onset for all 20 target brands. No missing values occurred. The final data sets each included 10 single skin conductance or heart rate values for liked brand names and further 10 single skin conductance or heart rate values for disliked brand names. These two separate data sets were then subject to statistical analysis in terms of repeated measures ANOVA to calculate condition main effect significance. Effect sizes were also calculated to better interpret statistical findings.

## Results

### 1. Startle reflex modulation

**Figure 2:**
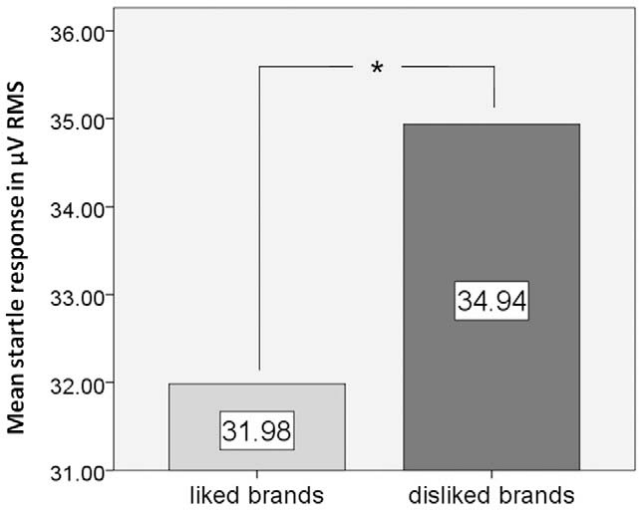
Bar diagram showing mean eye blink amplitudes related to both conditions of interest, visual presentations of liked and disliked brand names. Note that mean eye blink amplitude related to visual presentations of liked brand names was significantly smaller than mean eye blink amplitude related to visual presentations of disliked brand names. This reflects more positive motivation and emotion related to liked brand names compared to disliked brand names.

The mean eye blink amplitude during visual presentations of liked brand names across all study participants was 31.98 µV RMS (SD = 22.4). The mean eye blink amplitude related to visual presentations of disliked brand names across all study participants was 34.94 µV RMS (SD = 20.94). Repeated measures ANOVA revealed a significant condition main effect on eye blink amplitudes (F = 5.110, p = .035, η^2^ = .203). Figure 2 provides bar diagrams.

### 2. Skin conductance

**Figure 3:**
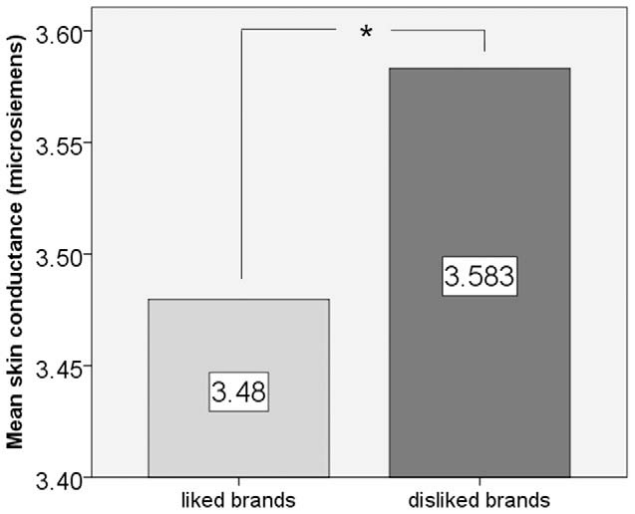
Bar diagram showing mean skin conductance amplitudes related to both conditions of interest, visual presentations of liked and disliked brand names. Note that mean skin conductance amplitude related to visual presentations of liked brand names was significantly smaller than mean skin conductance related to visual presentations of disliked brand names. Less skin conductance related to liked brand names might reflect greater relaxation due to positive associations.

We found a significantly reduced skin conductance value associated with visual presentations of liked brand names (mean = 3.48 microsiemens, SD = 2.02) compared to disliked brand names (mean = 3.583 microsiemens, SD = 2.08) Repeated measures ANOVA revealed a significant condition main effect (F = 12.581, p = .002, η^2^ = .386). Despite relatively high standard deviations because of inter individual differences, within-subject analysis revealed a significant condition main effect. Figure 3 shows a respective bar diagram.

### 3. Heart rate

**Figure 4:**
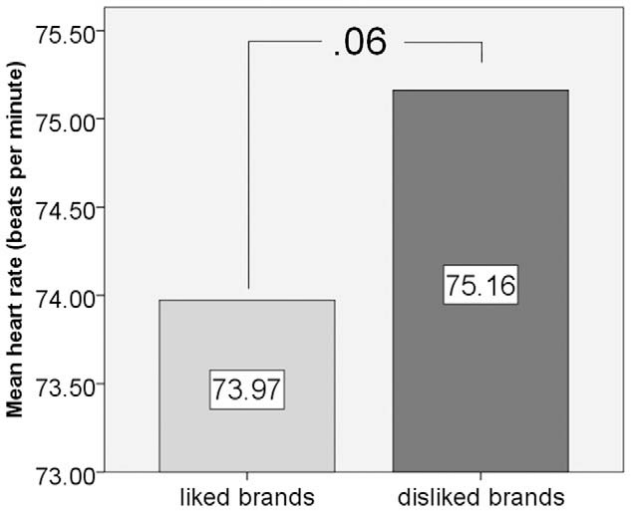
Bar diagram showing mean heart rate related to both conditions of interest, visual presentations of liked and disliked brand names. Note that mean heart rate related to visual presentations of liked brand names was smaller than mean heart rate related to visual presentations of disliked brand names. The increased heart rate related to disliked brand names fits the notion that visual presentation of disliked brand names elicits negative memories.

We found a strong trend towards a significantly reduced heart rate associated with visual presentations of liked brand names (mean = 73.97 bpm, SD = 8.08) compared to disliked brand names (mean = 75.16 bpm, SD = 9.17). Repeated measures ANOVA revealed an almost significant condition main effect (F = 3.970, p = .060, η^2^ = .166). Figure 4 shows a respective bar diagram.

See table 1 displaying the mean values plus standard deviations and p-values of all three measures and both conditions.

**Table 1:** Descriptive summary of mean values, standard deviations and p-values (t-tests) of all three measurements for both conditions of interest, liked and disliked brand names.

Mean values	liked brand names	disliked brand names	p-value
*eye blink amplitude*	31.98 µV RMS (SD = 22.4)	34.94 µV RMS (SD = 20.95)	P = .035
*skin conductance*	3.48 µSiemens (SD = 2.02)	3.583 µSiemens (SD = 2.08)	P = .002
*heart rate*	73.97 bpm (SD = 8.08)	75.16 bpm (SD = 9.17)	P = .060

## Discussion

The present study is meant to contribute to a better understanding of emotion-related aspects of brand attitudes while simply reading brand names. Traditionally, attitudes have been measured via self-report [33]. We know that self report can be extremely biased to one or the other direction by various uncontrolled factors. To date, while some still believe in self-report scales to diagnose people's attitudes others have already been using implicit measures to overcome the problem that at least some aspects of an attitude are not open to introspection [34]. One of these measures is the so called Implicit Association Test (IAT), which has been used to evaluate attitudes without demanding explicit responses [35]–[38]. The IAT is solely based on a response-time-bias being possibly evident in two critical blocks within an IAT experiment. Although this method may represent a helpful approach in terms of objective measurements it still cannot disentangle cognition- from emotion-related information processing. It has already been mentioned in the introduction that cognitive aspects are easier to determine because cognition is tightly linked to language whereas emotion is rather vague. However, emotion aspects are important because emotion-related information processing has an influence on attitudes and drives human behaviour to a wide extent. The results of our study provide insight into new possibilities to reliably quantify emotion-related information with respect to both known dimensions valence and arousal. Our study revealed via separate objective measures that valence is significantly different between liked and disliked brand names and that also arousal is different between liked and disliked brand names. Our study thus may provide a new approach to measure emotion-related information processing for marketing-relevant questions of interest.

### 1. Startle reflex modulation

Before actually starting to discuss our findings regarding startle reflex modulation it should be mentioned that until now it still remains unclear what exactly modifies the magnitude of startle responses. However, according to most of the literature it can be summarised that emotion-related aspects of a foreground stimulus (also called lead stimulus) are most likely influencing eyeblink amplitudes as responses to acoustic startle stimulation. There are reports about various other influences as well, but these seem to depend on specific circumstances related to stimulation procedures and instructions given to participants. According to a review about the psychological significance of human startle eyeblink modification by [29], emotion- and attention-related processes change eyeblink amplitudes in case of lead stimulus intervals longer than 800 ms. Since we presented our visual target stimuli (brand names) for 5 s each with our startle probe occurring 4.2 s after their onset we can assume that emotion- and/or attention-related processes were the factors modifying eyeblink amplitudes in our study. Since we did not change any requirements related to attentional processes (the task was always to focus attention on nothing but the visually presented brand names) we can rule out any attention-related effects. Consequently, we interpret our startle reflex modulation findings in terms of emotion-related influences of liked and disliked brands.

The startle reflex modulation findings from the present study can be interpreted in two directions. First of all, they are suggested to provide empirical evidence that brand attitudes indeed do contain emotion-related aspects with a specific focus on valence. This first claim is based on numerous previous investigations using startle reflex modulation. By using all sorts of emotional foreground stimuli such as facial expressions, sounds [39] and images from the International Affective Picture System [40] it was demonstrated that the magnitude of the startle reflex indeed reflects emotion valence rather than just arousal [41]. As already mentioned, it was shown that even startle habituation does not affect the sensitivity of a startle response to be modified as a function of emotion valence. Clearly, negative emotion enhances a startle response whereas positive emotion reduces it [42], [26]. In our study, we found significantly smaller eye blink responses when being startled while viewing strongly liked brand names in contrast to strongly disliked brand names. It might be helpful to emphasise that the relatively high standard deviations in our study mirror great inter-subject variability related to EMG responses. However, statistical analysis was done on a within-subject basis and therefore inter-subject variability does not matter. It is concluded that our findings demonstrate that liked versus disliked brand names indeed elicit different emotions or degrees of pleasantness (valence) while being read. Secondly, the present study demonstrates that such emotion-related information underlying brand attitude can be quantified without demanding explicit statements about subjective preferences. The idea to quantify emotion-related information on a fine graded scale with respect to viewing brand names is intriguing and potentially leading to various useful applications. By using startle reflex modulation it was recently shown that emotion-related information varies as a function of intake of different foods [24]. The authors found that eating chocolate compared to ice cream and yoghurt elicited the most positive emotion (highest degree of pleasantness) in female study participants without asking them about their feelings during food intake. Another study about virtual walks through urban environments demonstrated that an urban district with a high median real estate price elicits smaller eye blink amplitudes than a district with a low median real estate price [25]. Thus, it seems that startle reflex modulation can be used to objectively measure emotion impact in various applied settings.

### 2. Skin conductance

The skin conductance data from the present study also demonstrate a clear sensitivity related to subjective brand preference. Skin conductance was markedly reduced in case of visual perception of liked brand names versus disliked brand names. Skin conductance is usually suggested to measure levels of arousal rather than emotion valence [43]. Strong emotional situations are associated with the release of acetylcholine within the sympathetic nervous system which gets active during high arousal. This release in turn increases eccrine sweat gland activity which finally increases skin conductance.

In the present study, skin conductance was significantly reduced in case of viewing liked brand names compared to viewing disliked brand names. It can therefore be concluded that visual perception of liked brand names elicited a more relaxing state compared to disliked brand names. Whereas our startle reflex modulation data reflect more positive emotion in association with liked brand names our skin conductance data add that liked brand names elicit a more relaxing state than disliked brand names.

### 3. Heart rate

It is known that heart rate in humans varies over time even in the absence of any changes in physical activity. Besides respiration rate having an influence on heart rate [44] it is activity within the autonomic nervous system which leads to heart rate variability. Whereas skin conductance is more likely to reflect levels of arousal changes in heart rate seem to include some aspects related to emotion valence [32]. However, the situation seems a bit ambiguous since increases in heart rate were found related to negative emotion, but under some circumstances also related to positive emotion. However, surprisingly enough, it was nicely demonstrated the event-related character of heart rate variability [32]. Even rapid changes in emotion content in the range of a few seconds resulted in significant heart rate changes in the range of about 1 beat per minute. In their study, viewing unpleasant pictures lead to significant decreases in heart rate. On the other hand, it was found that imagination of an unpleasant event increases heart rate. Their experiment included recall of previously presented sentences with personally related fear content [45]. We found a strong trend towards an increase in heart rate during visual presentation of known disliked brand names. We conclude that brand name presentation elicited personal experiences with these brands. Obviously, in case of disliked brand names the experiences were rather negative.

### 4. Final remarks

In particular, the significance of startle reflex modulation could be successfully demonstrated. Attitudes towards brands contain describable emotion aspects. Startle reflex modulation offers the opportunity to get insight into emotion-related aspects of brand attitude without demanding explicit responses. It overcomes the weaknesses traditional self-report measures of brand attitude have to deal with. It overcomes the issue that many responses to attitude questions may be measurement artefacts created simply because the question was asked [46]. Self reported emotions will always be the result of cognitive reflections. With regard to this drawback we want to create awareness about our theory of cognitive pollution. Direct questions about one's emotion are always cognitively polluted. Although this particular theory was not tested here we believe that future research will focus on that notion. Finally, we want to mention that startle reflex modulation may be introduced into any marketing-related research in the future, because it is a cheaper and a better method than any brain imaging method when it comes to quantify emotion on a fine graded scale. Also, product development and product evaluation can enormously profit from that.
